# Influence of Polymer Fibre Reinforcement on Concrete Anchor Breakout Failure Capacity

**DOI:** 10.3390/polym16152203

**Published:** 2024-08-02

**Authors:** Julia Spyra, Nikolaos Mellios, Michael Borttscheller, Panagiotis Spyridis

**Affiliations:** 1 Faculty of Architecture and Civil Engineering, TU Dortmund University, 44227 Dortmund, Germany; julia.spyra@tu-dortmund.de (J.S.); nikolaos.mellios@tu-dortmund.de (N.M.); 2 Ingenieurbüro Schülke Wiesmann | Tragwerksplanung, 44141 Dortmund, Germany; 3 BarChip EMEA Ltd., Dublin 24, Ireland; mborttscheller@barchip.com; 4 Concrete Structures and Infrastructures, University of Rostock, 18051 Rostock, Germany

**Keywords:** polymer fibre concrete, polymer fibre reinforcement, fastening technology, anchorages, concrete breakout

## Abstract

With the increasing use of fibre-reinforced concrete, e.g., in industrial floor and tunnel construction, the associated fastening technology in this material has increasingly become the focus of scientific attention in recent years. Over 25 years ago, design and assessment guidelines for anchoring systems in reinforced concrete were established, which have since evolved into comprehensive regulatory standards. However, these standards only address plain and rebar-reinforced concrete as anchoring bases, neglecting fibre-reinforced concrete. The design of anchorage systems in fibre-reinforced concrete has not yet been standardised. Recent studies and product certifications accounting for steel fibre reinforcement are now seeing their way to publication, supported by a fair amount of scientific research studies. This paper aims to elucidate the effects of polymer fibre reinforcement in this application through a systematic investigation. Experimental studies were conducted to evaluate the system’s load-bearing behaviour failing with concrete breakouts under tensile loading. By incorporating the determined material properties of polymer fibre-reinforced concrete and their mathematical interpretation, alternative model proposals are presented to assess concrete breakout resistance. The addition of polymer fibres significantly improves the load-bearing capacity and ductility of concrete under tensile loads, transforming its quasi-brittle response into a more ductile behaviour. Although the fibres had a minor impact on overall material strength, their influence on the tensile capacity of the anchors reveal a 15–20% increase in load resistance and up to a doubling of the failure displacements.

## 1. Introduction

Concrete with the addition of fibres is increasingly being used in the construction industry complementing or even replacing the usual steel-bar reinforcement. In the meantime, intensive research and committee activities have led to a sound and consenting knowledge of the material characteristics. This makes it possible to assess the advantages and disadvantages of this innovative building material in terms of practical and economic construction. In comparison to typical reinforced concrete, however, the applications and behaviour of anchorages are still little researched. A design and assessment guide for fasteners in reinforced concrete was published more than 25 years ago, and there are now comprehensive design standards in force; however, particularly with regard to the load-bearing behaviour of fasteners in fibre-reinforced concrete, the technical community is still in the process of delivering design and certification rules for this relatively new building material as an anchoring base.

The load-bearing capacity of anchors for use in industrial floors for fastening high racks and in tunnel construction for attaching suspended ceilings or technical equipment is particularly significant, as these are frequent areas of application for fibre-reinforced concrete. Although steel fibre-reinforcement effects have been researched to a certain extent (see [1,2,3,4,5,6,7,8,9]), polymer fibre reinforcement has not been consistently addressed. At the same time, macrosynthetic-fibre reinforced concrete has proven to be a material with very efficient mechanical properties, durability advantages (corrosion-free), energy absorption performance, cost and sustainability ratings [10,11,12]. This forms the focus on this paper. To this end, fasteners were tested in polymer fibre concretes with different mix designs and different fibre contents. An additionally produced reference concrete without fibres was used to compare how the load-bearing behaviour of fasteners in this type of concrete changes compared to that in fibre-reinforced concrete, and whether the addition of even small amounts of fibres can have a positive effect in terms of load-bearing capacity.

After an overview of the basics of fastening technology and fibre technology, this paper aims to identify possible factors influencing the anchoring of fastening elements in concrete with structural polymer fibres and their effects on load bearing and deformation behaviour. Furthermore, it attempts to identify suitable material-strength predictors for use in design equations. Based on a series of experimental investigations, the load-bearing behaviour of single anchors and its relationship with fibre content and the macroscopic tensile and compression material parameters are mathematically described, and recommendations for future design procedures are delivered.

## 2. Theoretical Background

### 2.1. Basic Principles of Fastening Technology

**Figure 1:**
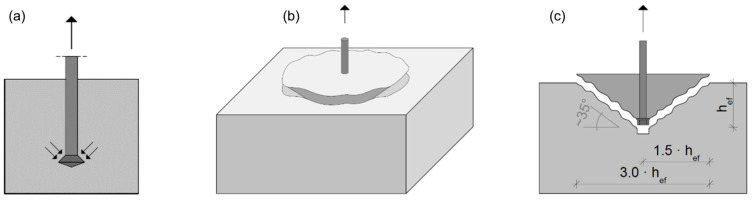
Mechanical interlock and concrete breakout formation (schematic representation acc. to [13,14]); (**a**): load transfer (black vectors) from external force to substrate for undercut anchors; (**b**): three-dimensional perspective of breakout failure in concrete specimen; (**c**): characteristic geometric properties of anchor breakout failure in cross-section.

In fastening technology, a distinction is made between three different types of anchoring mechanism, namely mechanical interlock, friction, and bond (the latter being a combination of adhesion and material interface cohesion). The principles of action differ fundamentally in the transmission of the centric tensile load into the concrete substrate, but a radial, cone-shaped stress distribution is commonly present. Headed bolts cast into concrete, such as the ones implemented in this work, create a resistance against tensile loads by means of form-locking with the head of the fastener, which describes the load transfer from the fastener to the anchor base by means of mechanical interlocking (Figure 1a). The anchor tensile force leads to local tensile stresses at the load introduction point in the anchor base or at the fastener itself. It is precisely at this point that the first radial cracks occur. These develop into macrocracks with increasing load and can ultimately lead to the total failure of a fastener by rupture of the concrete.

Characteristic of the “concrete breakout” failure mode, which is the failure objective of the tests in this work, is a cone-shaped concrete body teared away from the solid and stable concrete substrate in which the fastener is mounted (Figure 1b). The fracture cone has a depth that corresponds to the effective anchorage depth h_ef_, the angle of inclination of the cone surface is approximately 35° [13] and the diameter of the concrete breakout body is typically three times the effective anchorage depth (Figure 1c). The characteristic resistance is influenced by the concrete material strength, the condition of the concrete (cracked/uncracked) and the anchorage depth of the fastener, as well as by the existing axial and edge distances, and to a lesser extent by the anchor and head diameter for typical bolted connections, such as the ones investigated herein [13].

To estimate the mean capacity of a single-headed anchor in plain, uncracked concrete without edge influence, the well-established Concrete Capacity Design (CCD) method can be used according to [13,14] (and further explained in [15,16]), as expressed in Equation (1), where N_Ru,c,uncracked_ is the mean uncracked capacity (in N), 16.8 is a unitless calibration factor specific for the anchor type or product (for an anchor-located cracked concrete, a reduced k-factor applies), $h_{\mathrm{ef}}$ is the anchor embedment depth (the distance of the failure invitation point to the free surface in mm), and $f_{c}$ is the mean cylinder compressive strength, as a representative and practical value for the tensile capacity for typical concretes (in MPa) [13]. It is noted for clarity that Equations (1) and (2), as well as other equations derived thereof throughout the paper, are semi-empirical and dimensionally inconsistent with their input parameters.

For design purposes, the European Committee for Standardization [17] proposes the calculation of the characteristic resistance through Equation (2), where N_Rk,c_ stands for the characteristic resistance and $f_{\mathrm{ck}}$ is the characteristic cylinder compressive strength, and it recommends k = 12.7 (unitless) for cast-in headed studs in uncracked concrete.

(1) $$N_{\mathrm{Ru},c}\;=16.8\cdot {{\;h}_{\mathrm{ef}}}^{1.5}\cdot \sqrt{f_{c}}\;[N],$$

(2) $$N_{\mathrm{Rk},c\;}^{\;}=k\;\cdot {{\;h}_{\mathrm{ef}}}^{1.5}\cdot \sqrt{f_{\mathrm{ck}}}\;[N]$$

### 2.2. Current Knowledge on the Influence of Fibres in Concrete Anchorages

The beneficial effects of fibre reinforcement on the load-bearing capacity of concrete anchors have been documented to some extent, on the basis of steel fibre reinforcement. Research has explored those in various contexts, including post-installed [1,2,3,4], cast-in anchorage systems [5,6,7], and non-standard, high-performing concretes [8,9]. Studies have demonstrated that fibres effectively restrain crack initiation and propagation around anchors, preventing the formation of a concrete breakout cone, thus enhancing both the resistance and ductility of the system [5,11]. The structural response of steel fibre-reinforced concrete, and consequently its impact on anchor performance, is shown to be strongly influenced by several factors. A higher fibre content generally leads to improved load-bearing capacity [5,11]. Additionally, the fibre material, shape, and efficiency of the bond within the concrete matrix play a crucial role in the results [5]. A recent study also disclosed the effects of fibre orientation in relation to the anchor axis [8]. Extensive systematic research studies have examined the effects of diverse steel fibre-reinforcement parameters, including fibre type, content, and concrete strength (in both cracked and uncracked states) on headed stud anchors [4,5,11,12], indicating some correlation between the concrete material properties and the anchor load capacity. An undenied outcome is understandably that Equations (1) and (2) are not applicable anymore as such, due to the different correlations between the compressive and tensile strength. A further analysis of the previous literature on the topic can be found in [5,11], where also the gap in the international literature regarding polymer fibre-reinforced substrates can be identified.

## 3. Experimental Investigations

### Experimental Programme

Four different concrete mixes were analysed for this study, as shown in Table 1. The concrete mixes differ fundamentally in the expected strength of the concrete, the water-to-cement (w/c) ratio and the fibre content.

The tests were designed in accordance with the test methodology presented in [17], and the prediction equations for mean static tensile resistance provided in [13] were used as a baseline reference for unreinforced concrete, to ensure that all tests fail with concrete breakout. The desired type of failure of the anchors in the concrete was concrete breakout, in order to discuss the possible influence of the changed concrete properties caused by the fibres on the load-bearing behaviour of the fasteners in the best possible way afterwards. Four test repetitions were carried out for each concrete mix, and in all tests, the breakout failure was achieved. In addition to testing the axial tensile capacity of the anchors in polymer fibre-reinforced concrete, the following concrete properties were also tested in accordance with the specified standards: concrete compressive strength (cube and cylinder compression test [18]) and concrete tensile strength (splitting tensile test [19], three-point bending test [20]). The test setup required us to perform the compression, splitting tensile and bending tensile tests and to determine if the modulus of elasticity complies with the specifications of [21,22].

## 4. Materials

### 4.1. Concrete

**Figure 2:**
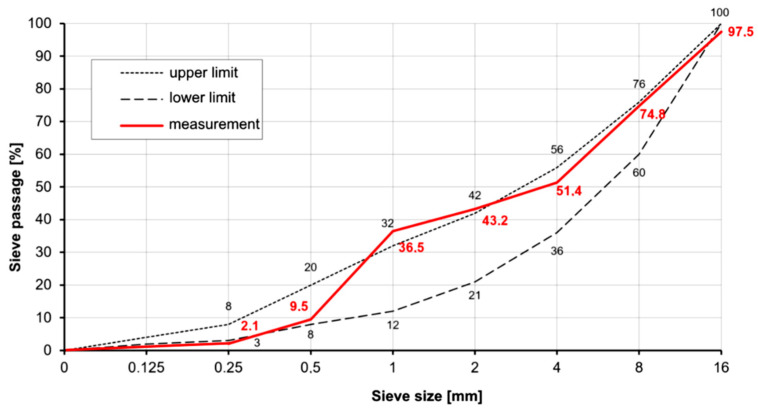
Sieve curve used for all specimen production.

The mix design relied on the author’s practical experience, with the principal aim to ensure that the proportion of coarse aggregate is kept as low as possible so that fibre agglomeration does not occur even with a higher fibre content [23]. The grading curve used for all concrete mixes produced can be found in Figure 2, and the mix details are in Table 2. An aggregate size between grading curves A and B (mean upper and lower limits from Figure 2) has proven to be a favourable grading curve range [5,8,23]. The maximum particle size used is d_g_ = 16 mm. Note that the addition of fibres increases the specific surface area of the mix constituents and thus also increases the water demand of the concrete [24]. To allow direct comparison for the experiments herein, all fibre reinforced specimens used the same mix—although in reality the mix shall be adjusted to optimally fit the polymer fibre content. The increased water demand had to be compensated by the addition of a polycarboxylate ether (PCE)-based MasterGlenium ACE 460 superplasticiser, at 0.55 L/ton. It was hence deemed suitable to use a different w/c ratio, which allowed for the same porosity and relatable concrete strength and avoided bleeding in high fibre contents. The cement type used was CEM I 42.5. The strength class C30/37 was targeted, as it is a commonplace choice in practice for polymer fibre concrete applications. To assess the consistency of the fresh concrete, the slump of the fresh fibre-reinforced concrete with polymer fibres was measured according to [24], and a soft consistency (F3) was determined for the mixtures bc4 and bc6 with slumps of 440 mm and 480 mm, respectively, whereas a plastic consistency (F2) was determined for the mixture bc8, with a slump of 380 mm. During production, three random samples were taken from each batch to confirm the actual fibre distribution and content using the washout test. The differences were in a tolerance of 1/1000, indicating that an even distribution of fibres was achieved.

#### 4.1.1. Fibre Reinforcement

BarChip48 polymer fibres from BarChip Inc. were used in this study. They are structural macrofibres made from virgin polypropylene, with a length of 48 mm, a Young’s Modulus of 12 GPa, and a rupture strength of 640 MPa. Their embossed surface enhances the bonding capabilities of load transfer to the concrete matrix. For structural applications with normal and high strength concrete, the fibre reinforced material classification is based on the post-cracking residual strength, by considering the characteristic flexural residual strength values that are relevant for serviceability (f_R1k_) and ultimate (f_R3k_) conditions [25]. BarChip48 polymer fibres can be used for structural applications according to [26] with suitable concrete recipes in cast-in-place and ready-mixed concrete applications with fiber contents of 4–10 kg/m^3^.

#### 4.1.2. Anchoring Elements

**Figure 3:**
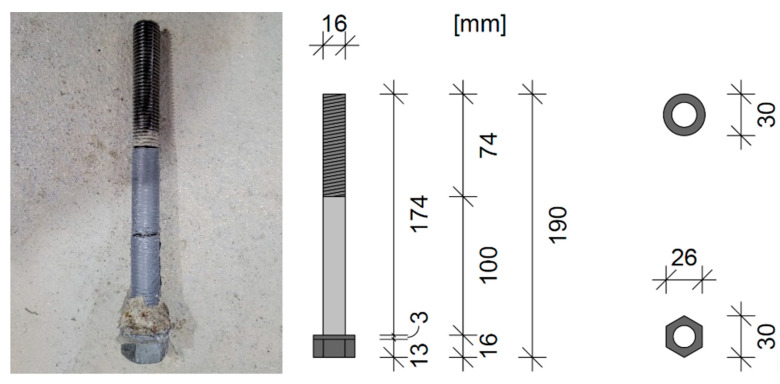
Headed bolt used; (**left**): bolt after use, (**right**): schematic representation of the bolt.

The anchors for the concrete breakout tests, which resemble the appearance and function of a headed bolt, were manufactured in-house and consist of 19 cm long M16 threaded rods with a passing HV hex-nut and round washer. All individual parts of the fastening element were assigned to strength class 12.9, which corresponds to a tensile strength of 1200 MPa and a yield strength of 1080 MPa, in order to rule out steel failure during the tests. The washer was fixed with hot glue to prevent displacement during the vibration process. The outer diameter of the washer was 30 mm and served to transfer the applied tensile force into the surrounding concrete via positive locking. The effective anchoring depth h_ef_ of the fasteners was 100 mm for all tests. In order to minimise additional anchoring of the threaded rod via friction, the embedded threaded part was covered with loosely wound adhesive tape (see Figure 3).

### 4.2. Experiment Setup and Execution

At the time of the tests, all test specimens had reached the 28-day strength. The cube- and beam-shaped test specimens were produced in a separate mould during concreting. The test cylinders required for the tests to determine the cylinder compressive strength and the splitting tensile strength were taken from the test slab in the form of drill cores parallel to the anchor axis after the completion of the tests, and they were ground down to 200 mm. A total of nine cores were taken for each concrete mix.

**Figure 4:**
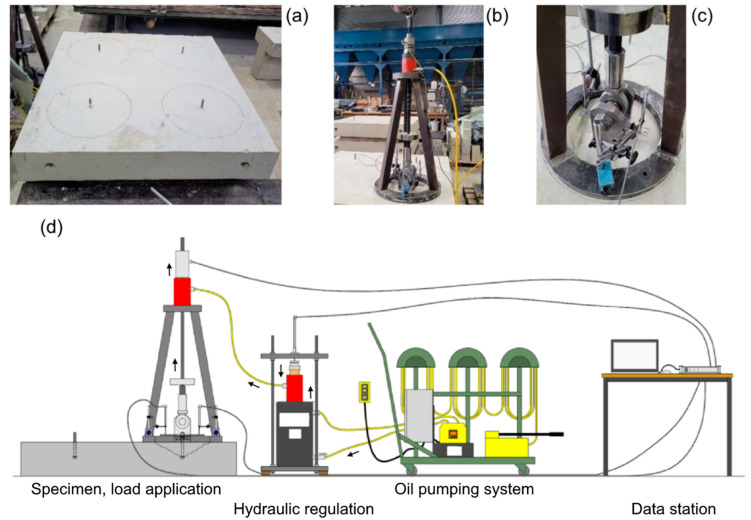
Aspects of the test setup: (**a**) specimen and counter support perimeter, (**b**) load cell and support frame, (**c**) load adapter and LVDT measurements, (**d**) schematic representation of experimental arrangement including hydraulic pressure flows.

**Figure 5:**
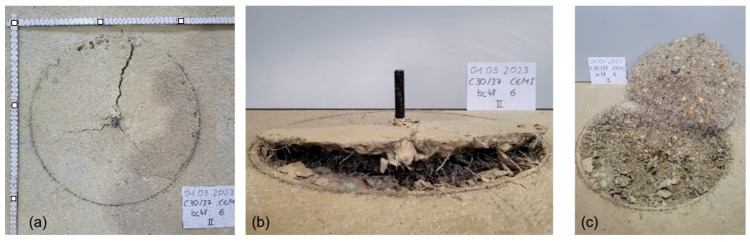
Indicative breakout failure taken on specimen bc6, Nr.2, on 01/03/2023 as indicated on the paper note. (**a**,**b**: test specimen top and side views after the end of the test, **c**: levered-out test specimen). A metric ruler illustrates the scales in (**a**), with marks at 200 mm.

Breakout tests were carried out on slab specimens measuring 160 cm × 160 cm × 25 cm. In order to prevent the influence of neighbouring anchors and to ensure unhindered symmetrical concrete breakout, a free radius greater than three times the anchoring depth h_ef_ was adopted, as seen in Figure 4. In particular, a ring-shaped support with an internal diameter of 41 cm was used as a counter support to the load application piston. The displacements of the anchor during pulling were measured with two linear variable differential transformers (LVDTs) fixed magnetically on this rigid ring. All relevant data were recorded using the Catman measurement software and the Spider8 measurement module (HBM) and subsequently analysed using spreadsheets. The data output included the test clock, the force, the loading rate, the values of the individual displacement transducers, and the resulting mean displacement. Figure 5 illustrates a representative breakout cone failure (in particular of test bc6_02).

## 5. Overview of Test Results

Concrete strength normalisation: The test slabs, cubes and beams had a concrete age of between 28 and 30 days at the time of testing; the tests with the cylinders were carried out with a concrete age of between 31 and 39 days. The characteristic values for the strengths and breakout loads are calculated as 5% quantiles of a non-negative logarithmic normal distribution in accordance with Appendix D of [25]. The experimental determination of the anchors’ load-bearing capacity was carried out on test specimens with varying strengths. In order to keep the tests performed in concrete with various compressive strengths comparable, every test result is normalised to a hypothetical cube strength of 45 MPa by multiplication with the normalising factor α = $\sqrt{{45/f}_{c}}$, where f_c_ is the recorded cube compressive strength. N_u,m_ describes the mean maximum breakout load of the respective test series and N_u,m,45_ means the associated normalised loads.

Characteristic values: The 5% quantile values of the normalised breaking loads are marked as N_R,ck,45_. In addition, the characteristic resistance of a cast-in headed bolt in non-cracked normalised concrete according to [5] is listed in the table as N_Rk,c_ for comparison. The results of all tests are summarised in Table 3.

Material variability: The coefficients of variation for the material tests came to a maximum of 9.5%, which is interpreted as pure material randomness, and to a lesser extent, testing uncertainties. In the breakout tests, the coefficient of variation reached a maximum of 5.1%, i.e., in a similar range and even lower in relation to the (unreinforced) material tests, indicating reliable test results, without additional uncertainties beyond typical material variance or testing complexity.

**Figure 6:**
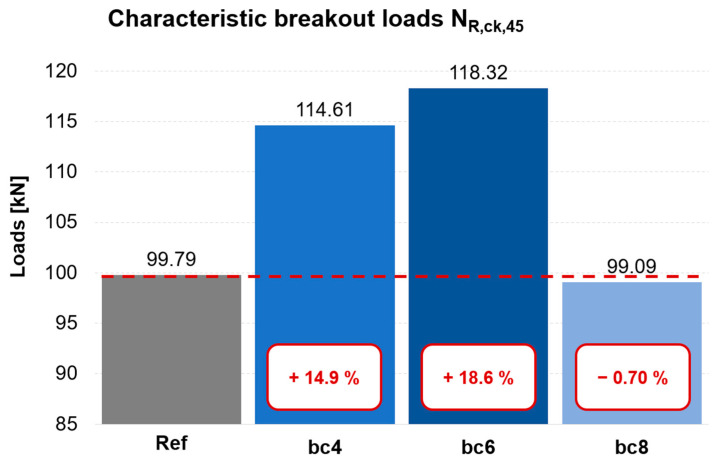
Characteristic breakout loads for all test series, indicating the change (absolute and percentual increase) of load resistance in polymer fibre-reinforced concrete, compared to plain concrete reference specimens; see Table 1 for explanation of Ref, bc4/6/8.

Effects of fibres: The comparison of the characteristic normalised breakout loads (N_R,ck,45_) of the polymer fibre test specimens in Figure 6 with those of the reference concrete without the addition of fibres shows that there is an increase in the maximum load in all cases. The load could be increased on average by up to 18.6%. The addition of polymer fibres has a positive effect on the load-bearing capacity of fastenings in concrete. However, no direct correlation of the fibre quantity on the breakout load was established. A hypothesis is that a fibre content of 8 kg/m^3^ in this specific case could not increase tensile strength results as compared to lower contents, given that the respective mix was not adjusted for the higher fibre dosageIn addition, a more ductile component failure with resistance retention could also be observed in the breakout tests compared to the reference test specimens.

**Figure 7:**
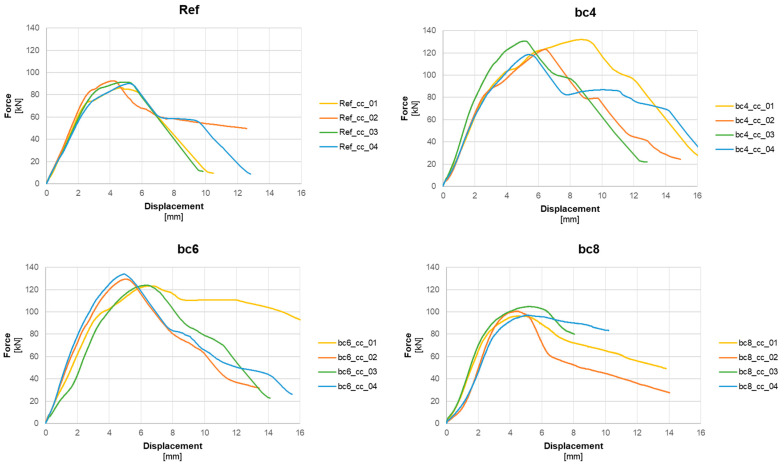
Load–displacement diagrams; see Table 1 for explanation of Ref, bc4/6/8.

Load–displacement performance: The load–displacement diagrams in Figure 7 illustrate the load-bearing performance of the different concrete mixes. The diagrams provide a comparative overview, confirming that the polymer fibre concrete (bc6 mix) exhibited the highest failure loads, while the reference mix showed the lowest failure loads. Furthermore, the highest deformability and post-failure retention support the anticipated result that fibre reinforcement enhances the overall robustness and ductility of the anchors. The bc8 tests do not show a significant difference from the reference tests in terms of failure displacements, which remain in the range of 4 to 6 mm. The maximum displacements at failure are observed for one of the bc4 tests at 8.5 mm. Both the failure loads and displacements share very common statistical values (mean values, standard deviations) for Ref and bc8; this implies that the higher amount of 8 kg/m^3^ did not substantially contribute but neither compromised the material performance. The composite material’s structural properties rely here mostly on the concrete matrix, which would need an optimisation to allow enhancement by higher fibre dosages.

## 6. Statistical Interpretations and Predictive Modelling

Correlation diagrams are used to measure the strength of the statistical relationships between the determined mean excavation loads N_u,m_ and the various concrete properties. The linear regression analysis first identifies the relationship between the breakout loads and the individual concrete properties. In the next step, the information obtained is used to develop a design concept with which the breakout loads can be predicted as accurately as possible. For the statistical assessment, the absolute residuals and the sum of the squared residuals (SSR) is also determined. In addition, the regression standard error (SE) is used to quantify the extent to which the measured results deviate vertically from the estimate function [27]—see Table 6.

The material-strength linear regression represents the correlation between the excavation loads and the concrete properties according to the general Equation (3). Table 4 lists the functions y of the regression lines and the coefficients of determination R^2^. The variable x describes the respective material-strength parameter in the line.

(3) $${\hat{N}}_{u,m}=y\;\cdot {h_{\mathrm{ef}}}^{1.5}$$

where y is a function according to Table 4 and h_ef_ is the effective embedment depth [mm].

**Figure 8:**
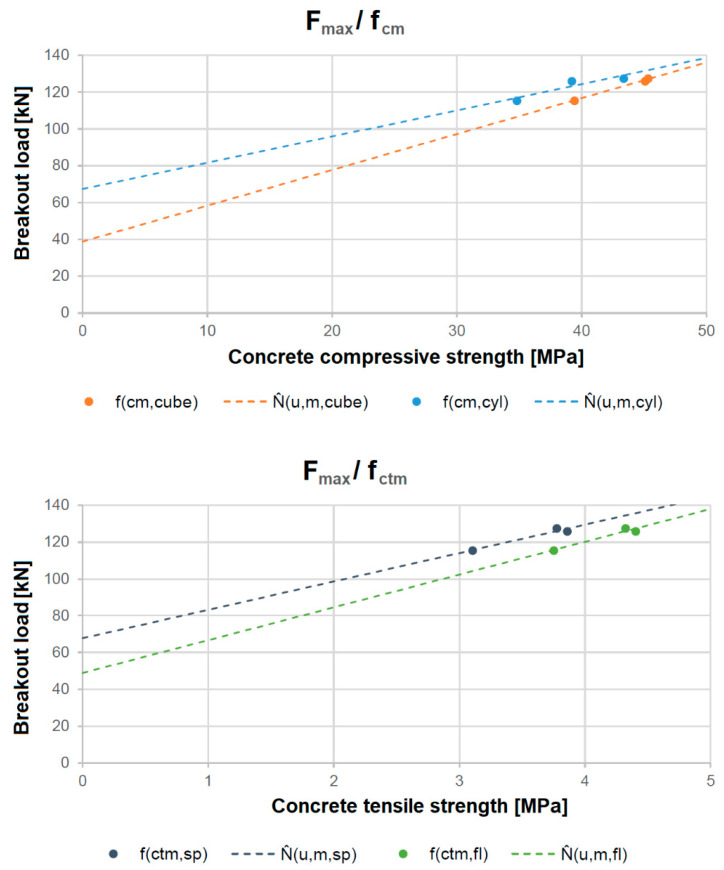
Linear regression between the concrete compressive strength (**top**) and tensile strength (**bottom**) and the actual mean failure loads.

Figure 8 illustrates the regression lines for the correlation of the breakout load with the cube and cylinder compressive strength (top) and the splitting and bending tensile strength (bottom). The calculation results in Table 5 show that the average errors made when using the functions described are between 0.5 and 2.0. The fewest errors are made when using the cube compressive strength as an estimate. It should be noted that this is not deemed applicable for low concrete strengths, i.e., below a strength class of C12/15.

**Figure 9:**
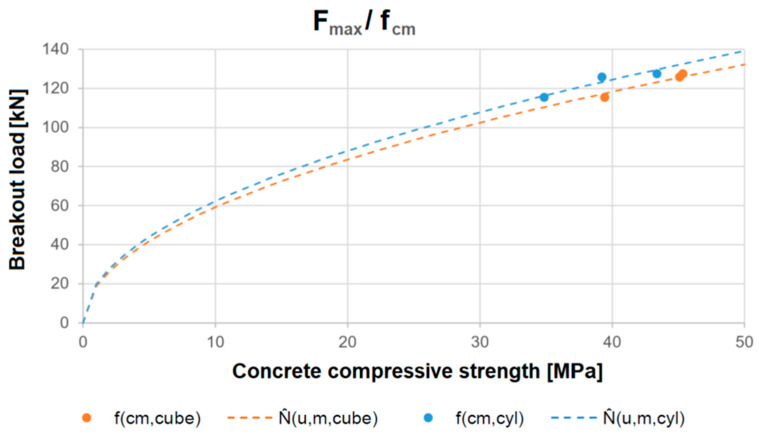

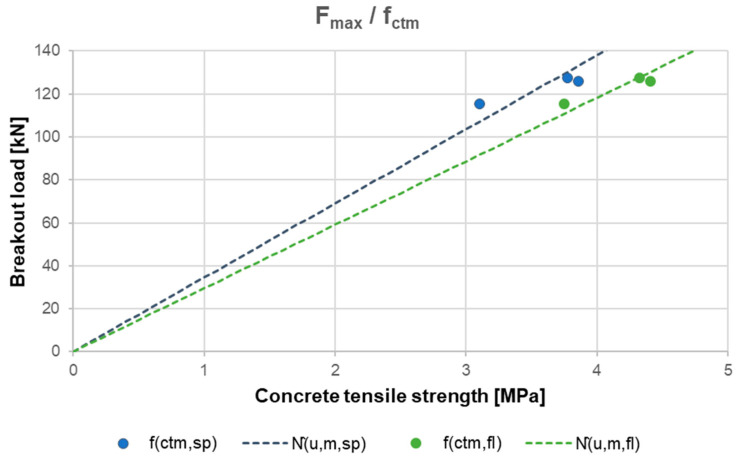
Adjusted CCD prediction equations, with reference to the concrete compressive strength (**top**) and the tensile strength parameters (**bottom**).

Furthermore, based on the CCD method (Equations (1) and (2)), the functions are used in the equation to determine the characteristic resistance of a fastening element, according to [3]. To approximate the anchor resistance, an adjustment of the CCD method per Equation (4) is used. In this case, the square root of the compressive strength is used according to the original CCD concept, but the tensile strength parameters of the concrete are also examined comparatively. Table 3 shows that the calculated characteristic resistances are significantly lower than the characteristic values from the tests. The factor k_1_ is calibrated using the mean values of the test results with the adjusted formula to capture the failure loads, as seen in Equations (5) and (6), with the results illustrated in Figure 9. The right part of Table 5 shows the prediction results using the adjusted CCD approach, and the test values are also provided in the left column for comparison. The least variations are found when estimating by use of the cube compressive strength. The average errors associated with using the adjusted CCD approach are in the range of 1.5 kN for the compression parameters, but the prediction errors are higher when directly using tensile parameters. As with the material-strength linear regression, it must be noted that this model is not considered applicable in the low strength range. As with the material-strength linear regression, it must be noted that this model is not considered applicable in the low strength range.

(4) $$k_{1}=\frac{N_{u,m}}{f\cdot {\;h}_{\mathrm{ef}}^{1.5}}$$

where N_u,m_: ultimate breakout load [N]; f: concrete strength from the various material characterisation modes [MPa]; h_ef_: effective embedment depth [mm].

(5) $${\hat{N}}_{u,m,\mathrm{cube}}\;=18.70\;\cdot \;\sqrt{f_{\mathrm{cm},\mathrm{cube}}}\;\cdot \;h_{\mathrm{ef}}^{1.5}\;[N]$$

(6) $${\hat{N}}_{u,m,\mathrm{cyl}}\;=19.68\;\cdot \;\sqrt{f_{\mathrm{cm},\mathrm{cyl}}}\;\cdot \;h_{\mathrm{ef}}^{1.5}\;[N]$$

(7) $${\hat{N}}_{u,m,\mathrm{cyl}}\;=34.51\;f_{\mathrm{ctm},\mathrm{sp}}\;\cdot \;h_{\mathrm{ef}}^{1.5}\;[N]$$

(8) $${\hat{N}}_{u,m,\mathrm{cyl}}\;=29.54\;f_{\mathrm{ctm},\mathrm{fl}}\;\cdot \;h_{\mathrm{ef}}^{1.5}\;[N]$$

where N_u,m_: ultimate breakout load [N]; f_cm,cube_: concrete cube compressive strength [MPa]; f_cm,cyl_: concrete cylinder compressive strength [MPa]; f_ctm,sp_: concrete splitting tensile strength [MPa]; f_ctm,fl_: concrete flexural tensile strength [MPa]; h_ef_: effective embedment depth [mm].

Table 6 lists all functions including their coefficients of determination, the mean residuals and the standard errors. In addition, the quality of the models is assessed in the last two columns, based on the ranking of their performance with all accuracy metrics. The classification is performed within the predicting method linear regression/adjusted CCD (M) and compared with all other methods (overall—O). The functions are ranked I to IIV and 1 to 8, respectively. The best-performing functions are based on the cube compressive strength, followed by the splitting tensile strength and the flexural tensile strength. Interestingly, whereas the cube compression appears to be the best predictor when considering the coefficient of determination (R^2^), the cylinder compression is the worst fit. At the same time, replacing the square root of the compressive strength with the tensile strength parameters in the CCD formula adjustment yields high error values, but the coefficient of determination shows good prediction efficiency. The regression lines have generally higher coefficients of determination.

## 7. Conclusions

With the addition of polymer fibres in different quantities, a significant improvement of various properties can be achieved. This paper discloses an increased load-bearing capacity of anchors in polymer fibre concrete under tensile loads, which was observed in some of the test series. The post-cracking residual tensile strength of the concrete, which was witnessed in both the splitting tensile tests and the bending tensile tests, also shows that it is possible to retain loads beyond the failure point. The quasi-brittle response of normal concrete in anchor tests is hence transformed into a substantially ductile component behaviour. Specific conclusions of the study are provided below.

(1) A positive influence of the fibres was observed in breakout test series bc4 and bc6. The normalised tensile loads based on the cube compressive strength were higher for the fibre-reinforced concrete than for the reference concrete in these cases. Overall, the polymer fibre reinforcement contributed significantly to an increased breakout load in these cases.
(2) It is well known, that the addition of Polymer-Fibres has decisive influence on residual strength properties (post-ductile behaviour—toughness) and minor influence on the axial tensile, splitting and flexural tensile strength (LOP) at failure. Apart from the favourable anchor resistance and ductile post-cracking behaviour of the polymer fibre-reinforced concrete in tests bc4 and bc6, the other results were within the range of the values of the reference concrete. The increased strength of the polymer fibre concrete is mainly due to the different mix designs compared to the mix design of the reference concrete, which is assumed to also lead to an increased bond strength of the fibres and hence a slightly higher contribution thereof. A further optimised mix may allow stronger contribution of fibres in bc8.
(3) Based on the test results, and with various failure load-prediction approaches, it is possible to estimate the load-bearing capacity of fastening elements in polymer fibre concrete. The correlations with cube compressive strength have proven to be particularly accurate estimation parameters. Conversely, the compressive strength of concrete cylinders has shown to be less reliable as a predictor. Given that fibre reinforcement primarily influences the tensile properties, it is reasonable to focus on finding optimal correlations between the anchor breakout resistance in fibre-reinforced concrete and its splitting tensile strength as well as its flexural and residual tensile strength.
(4) Based on the tests carried out, no linear correlation could be established between the fibre content and the breakout load. An optimum fibre content may be below the maximum tested fibre content. Based on the experience gained, a hypothesis for future investigations is formed that high fibre contents may lead to optimal mechanical properties of anchorages, if the concrete mix is accordingly designed and produced.
(5) The results herein do not strongly support the benefits of using tensile parameters or the fibre content as a varying design parameter. However, it is anticipated that elaboration of a large, more comprehensive dataset of tests with various fibre-reinforcement types and quantities as well as matrix strength parameters will disclose such assessments with a higher degree of confidence. As a further study prompt, material performance metrics based on toughness test methods should be tested as a representative description of the strength contribution of macrosynthetic fibres for design purposes.
(6) It should be noted that the tendencies of an increased load-bearing capacity of fasteners in fibre-reinforced concrete are so far only taken from a small number of tests and should be verified in the future by more extensive tests with a broader coverage of testing parameters. Nevertheless, the addition of the reinforcing fibres has led to equal or greater load-bearing resistance at mean and at characteristic values for all test series, while the increased ductility of these anchorages confirms an enhancement of their performance in terms of structural safety.

The observation of an increased load-bearing capacity of anchors in polymer fibre concrete lays the foundation for more efficient structural connection techniques.

## Figures and Tables

**Table 1:** List of the concrete mixes used and designation of test series.

Designation	Concrete Class	w/c Ratio	Fibre Reinforcement	Fibre Content
Ref	C20/25	0.65	Baseline reference (unreinforced)	0 kg/m^3^
bc4	C30/37	0.50	Polymer fibres BarChip48,	4 kg/m^3^
bc6	C30/37	0.50	Polymer fibres BarChip48,	6 kg/m^3^
bc8	C30/37	0.50	Polymer fibres BarChip48	8 kg/m^3^

**Table 2:** Concrete mix proportions; see Table 1 for explanation of Ref, bc4/6/8.

Constituents Per m^3^			Ref	bc4/6/8
Surface moisture		[kg]	1	1
Added water		[kg]	171	172
Cement		[kg]	267	344
Superplasticiser		[L/ton]	-	0.55
Aggregates	8/16	[kg]	490	473
	2/8	[kg]	589	568
	0/2	[kg]	883	853
Pore volume		[m^3^]	0.015	0.015
Fibres		[kg]	-	4/6/8

**Table 3:** Summary of all test results (averaged per test series, st. dev. Stands for “standard deviation” per test series for the actual test results); see Table 1 for explanation of Ref, bc4/6/8.

Test Series	Compressive Strength		Tensile Strength		Breakout Resistance Loads				Failure Displacements
	f_cm,cube_	f_cm,cyl_	f_ctm,sp_	f_ctm,fl_	N_u,m_ (St. Dev.)	N_Rk,c_	N_u,m,45_	N_R,ck,45_	δ (St. Dev.)
	[MPa]	[MPa]	[MPa]	[MPa]	[kN]	[kN]	[kN]	[kN]	[mm]
**Ref**	32.8	28.1	3.00	3.6	90.02 (4.72)	85.13	105.52	99.79	4.7 (0.4)
**bc4**	45.1	39.2	3.85	4.4	126.02 (6.37)	114.71	125.88	114.61	6.4 (1.6)
**bc6**	45.3	43.3	3.75	4.3	127.63 (5.66)	118.74	127.21	118.32	5.7 (0.8)
**bc8**	39.4	34.8	3.10	3.8	99.61 (4.77)	92.72	106.44	99.09	4.8 (0.4)

**Table 4:** Values from correlation diagrams.

Correlation	Function y	R^2^
F_max_/f_cm,cube_	1.95x + 38.80	0.992
F_max_/f_cm,cyl_	1.42x + 67.43	0.859
F_max_/f_ctm,sp_	15.47x + 67.73	0.951
F_max_/f_ctm,fl_	17.86x + 48.83	0.944

**Table 5:** Material-strength linear regression error evaluation.

Test Series		Linear Regression Model				Adjusted CCD Model			
	N_u,m_	${\hat{N}}_{u,m,\mathrm{cube}}$	${\hat{N}}_{u,m,\mathrm{cyl}}$	${\hat{N}}_{u,m,\mathrm{cyl}}$	${\hat{N}}_{u,m,\mathrm{sp}}$	${\hat{N}}_{u,m,\mathrm{cube}}$	${\hat{N}}_{u,m,\mathrm{cyl}}$	${\hat{N}}_{u,m,\mathrm{sp}}$	${\hat{N}}_{u,m,\mathrm{fl}}$
	[kN]	[kN]	[kN]	[kN]	[kN]	[kN]	[kN]	[kN]	[kN]
bc4	126.02	126.64	126.64	123.19	127.28	125.60	125.60	132.85	129.96
bc6	127.63	127.03	127.03	129.02	125.73	125.88	125.88	129.40	127.01
bc8	115.56	115.54	115.54	116.93	115.68	117.40	117.40	106.97	112.24

**Table 6:** Comparison of the predictive model performances.

Model	Function	R^2^	SE		Model Performance	
		[-]	[kN]	[%]	M	O
Linear regression	$(1.95\;\cdot \;f_{\mathrm{cm},\mathrm{cube}}+38.78)\;\cdot \;h_{\mathrm{ef}}^{1.5}$	0.992	0.86	0.7	I	1
	$(1.42\;\cdot \;f_{\mathrm{cm},\mathrm{cyl}}+67.43)\;\cdot \;h_{\mathrm{ef}}^{1.5}$	0.859	3.44	2.8	IV	8
	$(15.47\;\cdot \;f_{\mathrm{ctm},\mathrm{sp}}+67.73)\;\cdot \;h_{\mathrm{ef}}^{1.5}$	0.951	2.28	1.9	II	3
	$(17.86\;\cdot \;f_{\mathrm{ctm},\mathrm{fl}}+48.83)\;\cdot \;h_{\mathrm{ef}}^{1.5}$	0.944	2.69	2.2	III	4
Adjusted CCD	18.70 · $\sqrt{f_{\mathrm{cm},\mathrm{cube}}}$ $\cdot \;h_{\mathrm{ef}}^{1.5}$	0.991	2.57	2.1	I	2
	19.68 · $\sqrt{f_{\mathrm{cm},\mathrm{cyl}}}$ $\cdot \;h_{\mathrm{ef}}^{1.5}$	0.873	3.41	2.8	IV	7
	34.51 · $f_{\mathrm{ctm},\mathrm{sp}}\;\cdot \;h_{\mathrm{ef}}^{1.5}$	0.941	11.12	9.0	II	5
	29.54 · $f_{\mathrm{ctm},\mathrm{fl}}\;\cdot \;h_{\mathrm{ef}}^{1.5}$	0.924	5.19	4.2	III	6

## Data Availability

Data are contained within the article.
